# Characterization and outcome of post-transplant lymphoproliferative disorders within a collaborative study

**DOI:** 10.3389/fonc.2023.1208028

**Published:** 2023-06-23

**Authors:** Philipp Lückemeier, Aleksandar Radujkovic, Udo Holtick, Lars Kurch, Astrid Monecke, Uwe Platzbecker, Marco Herling, Sabine Kayser

**Affiliations:** ^1^Department of Hematology, Cellular Therapy, and Hemostaseology, University Hospital Leipzig, Leipzig, Germany; ^2^Internal Medicine V, University Hospital Heidelberg, Heidelberg, Germany; ^3^Department I of Internal Medicine, Medical Faculty and University Hospital of Cologne, University of Cologne, Cologne, Germany; ^4^Department of Nuclear Medicine, University Hospital Leipzig, Leipzig, Germany; ^5^Department of Pathology, University Hospital Leipzig, Leipzig, Germany; ^6^NCT Trial Center, National Center of Tumor Diseases, German Cancer Research Center (DKFZ), Heidelberg, Germany; ^7^Institute of Transfusion Medicine and Immunology, Medical Faculty Mannheim, Heidelberg University, Germany

**Keywords:** PTLD, Epstein - Barr virus, allogeneic hematopoietic stem cell transplantation, solid organ transplantation, outcome

## Abstract

**Background:**

Post-transplant lymphoproliferative disorders (PTLD) are heterogeneous lymphoid disorders ranging from indolent polyclonal proliferations to aggressive lymphomas that can arise after solid organ transplantation (SOT) and allogeneic hematopoietic transplantation (allo-HSCT).

**Methods:**

In this multi-center retrospective study, we compare patient characteristics, therapies, and outcomes of PTLD after allo-HSCT and SOT. Twenty-five patients (15 after allo-HSCT and 10 after SOT) were identified who developed PTLD between 2008 and 2022.

**Results:**

Median age (57 years; range, 29-74 years) and baseline characteristics were comparable between the two groups (allo-HSCT vs SOT), but median onset of PTLD was markedly shorter after allo-HSCT (2 months vs. 99 months, P<0.001). Treatment regimens were heterogeneous, with reduction of immunosuppression in combination with rituximab being the most common first-line treatment strategy in both cohorts (allo-HSCT: 66%; SOT: 80%). The overall response rate was lower in the allo-HSCT (67%) as compared to the SOT group (100%). Consequently, the overall survival (OS) trended towards a worse outcome for the allo-HSCT group (1-year OS: 54% vs. 78%; P=0.58). We identified PTLD onset ≤150 days in the allo-HSCT (P=0.046) and ECOG >2 in the SOT group (P=0.03) as prognostic factors for lower OS.

**Conclusion:**

PTLD cases present heterogeneously and pose unique challenges after both types of allogeneic transplantation.

## Introduction

Post-transplant lymphoproliferative disorders (PTLDs) are a heterogeneous group of lymphoid or plasmocytic proliferations occurring after transplantation. The new World Health Organization classification of hematolymphoid tumors categorizes them as any hyperplasia or lymphoma arising in the immune deficiency/dysregulation setting post-transplantation (1). They are associated with iatrogenic immunosuppression and Epstein-Barr virus (EBV) in recipients of solid-organ transplant (SOT) or hematopoietic stem cell transplantation (allo-HSCT) (2). EBV-association is particularly pronounced in PTLD after allo-HSCT (~83%) as compared to cases after SOT (~33%) (3), often coincides with rapidly rising EBV DNAemia and mostly occurs within 100 days after allo-HSCT (4). While PTLDs are rare with an incidence of 1%-3.2% in the allo-HSCT setting, their incidence has been increasing alongside the number of transplantations, unrelated or HLA-mismatched related donors, selective T-cell depletion, antithymocyte globulin use and donor as well as recipient age (4–7). The risk of PTLD increases in patients with two or more risk factors (8). Thus, prospective monitoring of EBV activation and early treatment intervention seems to be reasonable in patients with elevated risk of PTLD after allo-HSCT. Clinically, extra-nodal disease is common, including 10%-15% presenting with central nervous system (CNS) disease (9). Despite improvements in treatment, PTLD remains one of the most life-threatening complications of allo-HSCT with an overall survival (OS) rate of ~50% after 6 years and a PTLD-related mortality rate of ~30% in the first year after diagnosis (3, 4). Standard risk-stratified therapy in patients with PTLDs after SOT consists of rituximab with or without chemotherapy (10, 11). Non-destructive PTLDs, also termed early lesions, tend to regress with reduction of immunosuppression (RIS) (12). Their prognosis seems to be excellent, if immune suppression reduction can be achieved without graft rejection. However, elevated lactate dehydrogenase, organ dysfunction, multi-organ involvement, advanced stage, bulky disease, and older age have been reported to be associated with a lack of response to decreased immune suppression (13, 14).

To address the need for standardization in patients with PTLDs after allo-HSCT, the Sixth European Conference on Infections in Leukemia (ECIL-6) published guidelines for the management of these patients (15). However, randomized controlled trials are sparse and treatment strategies remain to be standardized.

The aim of this retrospective study from three academic centers was to evaluate the characteristics and outcomes of PTLD in adult patients after allo-HSCT in comparison to PTLD after SOT.

## Methods

### Patients and treatment

Information on 25 adult patients with PTLD diagnosed between 2008 and 2022 (2008-2015, n=8; after 2015, n=17) was collected within a multicenter cohort (University Hospital of Leipzig, n=18; University Hospital Heidelberg, n=5; University Hospital Cologne, n=2). To identify patients, keyword searches including ‘PTLD’, ‘post-transplant’, and ‘lymphoproliferative’ were performed on all doctor’s letters and coded diagnoses of patients that visited the centers since 2008. Detailed case report forms (including information on baseline characteristics, chemotherapy, allo-HSCT, response, and survival) were collected from all participating centers. Inclusion criteria were adult patients with a confirmed diagnosis of PTLD following allo-HSCT or SOT. All patients who fulfilled these criteria were included by the participating groups/institutions, respectively.

### Response assessment

Morphologic response was routinely assessed by computed tomography (CT) scans or magnetic resonance imaging (MRI). Disappearance of lymphoma lesions is termed as complete response (CR) and regression of at least 50% as partial response (PR). Metabolic imaging by positron emission tomography (PET) using the radiotracer 18-fluorodeoxyglucose (^18^F-FDG) was capable of further distinguishing residual tumor tissue based on metabolic response. The Deauville score (DS) was used to graduate metabolic response in interim and end-of-treatment ^18^F-FDG PET scans (16).

### Statistical analysis

Survival endpoints including overall survival (OS) and relapse-free survival (RFS) were defined according to the revised recommendations of the International Harmonization Project in Lymphoma and the 2014 Lugano classification (16, 17). Comparisons of characteristics of patients with PTLD after allo-HSCT as compared to those after SOT were performed with the t-test for continuous variables (age), the Mann-Whitney test (e.g. for ECOG) and Fisher’s exact test (for cause of transplantation, sex) for categorical variables. Log-rank tests were employed to compare survival curves between groups. Median follow-up was calculated using the reverse Kaplan-Meier method. To identify prognostic variables with respect to outcome, a univariate analysis of preselected factors was performed. Variables included age, ECOG, IPI, and PTLD onset. All statistical analyses were performed with GraphPad Prism 7.

## Results

### Study cohort

Overall demographic and clinical data were collected from 25 patients diagnosed with PTLD between 2008 and 2022. Of those, 15 patients were diagnosed with PTLD after allo-HSCT and 10 patients with PTLD after SOT (6 liver, 3 kidney, 1 kidney and pancreas). There was no statistical difference in median age between the two groups (**Table 1**; P=0.70). In the allo-HSCT group, 11 of 15 (73%) patients were male as compared to 6 of 10 (60%) patients in the SOT group (P=0.67; **Table 1**). Ten (67%) patients in the allo-HSCT group were transplanted due to a hematological malignancy, whereas only one patient (10%) in the SOT group received a transplant due to a malignancy (hepato-cellular carcinoma; P=0.012). EBV association was detected in 13 of 15 (87%) patients in the allo-HSCT group and in 4 of 10 (40%) patients in the SOT group (P=0.03). Of the 14 allo-HSCT patients still receiving immunosuppressive agents at PTLD onset, 10 (71%) received cyclosporine, three (21%) received tacrolimus, and two (14%) received mycophenolic acid (one patient in conjunction with cyclosporine, ruxolitinib, and methylprednisolone). In the SOT group, all patients received immunosuppressive agents at PTLD onset: 8 (80%) patients received tacrolimus and the two other patients (20%) received cyclosporine. Moreover, 8 (80%) SOT patients additionally received mycophenolic acid and one SOT patient (10%) additionally received everolimus. Further baseline characteristics are summarized in **Table 1**.

**Table 1:** Baseline characteristics of patients with post-transplant lymphoproliferative disorders (PTLD) at onset of PTLD.

Patient ID	Age^a^	Sex	Tx type	Indication for Tx	Previous anthracycline- containing treatments	Conditioning	Time from Tx (months/yrs)^a^	Histology^a^	EBV+ (Lymphoma / Blood)	Ann Arbor stage^a^	ECOG^a^	IPI^a^
PTLD after HSCT:												
HSCT1	36	m	2nMMUD	sAML	1x 7 + 3, 1x IDA-FLAG	MA	2(0.2)	monom, DLBCL	pos/pos	n/a	3	n/a
HSCT2	65	m	MUD	AML	1x 7 + 3, 1x HAM	NMA	2(0.2)	n/a	na/pos	.	4	4
HSCT3	46	m	MUD	sAA	none	NMA	0(0)	n/a	na/pos	.	3	3
HSCT4	59	m	MUD	PCL	1x CAD	NMA	6(0.5)	n/a	na/pos	IV	3	3
HSCT5	61	m	MMUD	AML	2x 7 + 3, 1x HAM	MA	2(0.2)	n/a	na/pos	n/a	3	n/a
HSCT6	32	m	MUD	sAA	none	NMA	8(0.7)	monom, DLBCL	pos/pos	I	1	0
HSCT7	64	W	2^nd^ MUD	CNL	1x Mito-FLAG, 1x IDA-FLAG	NMA	4(0.3)	monom, DLBCL	pos/pos	IV	3	4
HSCT8	48	W	MUD	cALL	treatment within the GMALL 07/2003 trial	MA	2(0.2)	monom, MM	neg/pos	IV	3	3
HSCT9	64	m	AMMUD	sAML	2x 7 + 3	NMA	58(4.8)	monom Burkitt	neg/neg	IV	1	3
HSCT10	60	m	MUD	sAA	none	MA	1(0.1)	polymorphic	pos/pos	IV	2	3
HSCT11	61	m	MUD	sAA	none	MA	1(0.1)	n/a	na/pos	I	1	2
HSCT12	57	m	2nd MUD	T-ALL&AML	1x 7 + 3	MA	2(0.2)	monom, DLBCL	pos/pos	IV	2	3
HSCT13	47	m	MUD	Metachromatic leukodystrophy	none	NMA	2(0.2)	monom, DLBCL	pos/pos	.	2	3
HSCT14	50	w	MUD	Multiple Myeloma	3x TAD, 1x CAD	NMA	81 (6.7)	cHL	pos/na	I	0	0
HSCT15	67	w	MRD	MDS EB2	none	NMA	3(0.3)	monom., T-LBL	neg/neg	IV	2	5
**Median/%**	**59**	**73%**	**93% U**	**67% malignancies**	**60% anthracyclines**		**2(0.17)**	**80% monom.**	**87% assoc.**	**IV**	**2**	**3**
PTLD after SOT:												
SOT1	53	W	Kidney	CKD, Fabry disease			133(11.1)	monom, DLBCL	neg/na	I	1	0
SOT2	31	m	Kidney	Monolateral renal agenesis			118(9.8)	monom, DLBCL	neg/neg	IV	3	3
SOT3	29	m	Liver	PSC, autoimmune hepatitis			70 (5.8)	monom, DLBCL	pos/neg	IV	1	3
SOT4	74	W	Liver	Autoimmune hepatitis			80 (6.7)	polymorphic	pos/pos	IV	3	4
SOT5	67	W	Kidney	CKD, chr. glomerulonephritis			233 (19.4)	monom, DLBCL	neg/na	IV	2	4
SOT6	57	m	Liver	Alcoholic cirrhosis			4 (0.3)	monom, PBL	pos/pos	I	2	1
SOT7	54	m	Liver	HCC, alcoholic cirrhosis			44 (3.7)	monom, DLBCL	neg/neg	IV	2	3
SOT8	67	m	Liver	Alcoholic cirrhosis			132 (11)	monom, DLBCL	neg/na	I	2	3
SOT9	45	m	Kidney & Pancreas	CKD, T1D			132 (11)	polymorphic	neg/neg	IV	2	4
SOT10	48	W	Liver	Alcoholic cirrhosis			5 (0.4)	polymorphic	pos/neg	IV	2	4
**Median/%**	**54**	**60% / m**		**10% malignancies**			**99 (8.3)**	**70% monom.**	**40% assoc.**	**IV**	**2**	**3**

^a^Since/at diagnosis of PTLD, respectively. ^b^Where applicable (*i.e.*, CD20^+^, EBV-associated, IS still ongoing).

7+3, cytarabine and daunorubicin; (s)AML, (secondary) acute myeloid leukemia; assoc., associated; sAA, severe aplastic anemia; CAD, cyclophosphamide, doxorubicin, and dexamethasone; cALL, common acute lymphoblastic leukemia; cHL, classic Hodgkin lymphoma; CKD, chronic kidney disease; CNL, Chronic neutrophilic leukemia; DLBCL, diffuse large B-cell lymphoma; GMALL07/2003, study protocol containing daunorubicin and doxorubicin; HCC, Hepatocellular carcinoma; IDA-FLAG, idarubicin, fludarabine, cytarabine, G-CSF; Mito-FLAG, mitoxantrone, fludarabine, cytarabine, G-CSF; monom., monomorphic; PBL, Plasmablastic lymphoma; PCL, plasma cell leukemia; PD, Progressive disease; Pola, Polatuzumab-Vedotin; PSC, Primary sclerosing cholangitis; T-LBL, T-lymphoblastic lymphoma; T1D, Type 1 diabetes; TAD, thalidomide, doxorubicin, Dexa; T-ALL, T-acute lymphoblastic leukemia.

### Treatment prior to transplantation

Nine of ten (90%, 60% of the total allo-HSCT group) patients with malignancies in the allo-HSCT group had received an anthracycline-containing chemotherapy prior to transplantation as compared to none in the SOT group. In the allo-HSCT group, six (40%) patients received myeloablative (MA) and nine (60%) patients received non-myeloablative (NMA) conditioning regimens.

### Histological classification of PTLDs

In five of 15 (33%) patients of the allo-HSCT group, PTLD was diagnosed merely by EBV transcripts in peripheral blood (PB) and concomitant lymphadenopathy. Histological confirmation of PTLD was omitted in these cases due to a poor performance status [ECOG ≥3 in patients HSCT2-5 (**Table 1**)] or difficult accessibility of the lesion [hilar lymphadenopathy, patient HSCT11 (**Table 1**)]. All PTLD cases in the SOT group were histologically confirmed (18). The predominant classification of PTLD in both groups was monomorphic PTLD (allo-HSCT group, n=8/10, 80%; SOT group, n=7/10, 70%), one case of classic Hodgkin-Lymphoma like PTLD was identified (allo-HSCT group, n=1; 10%); all others had a polymorphic PTLD (allo-HSCT, n=1, 10%; SOT, n=3; 30%).

In the SOT group, 4 of 10 (40%) PTLD patients showed EBV-positivity in the lymphoma tissue. Importantly, of those, only two (50%) had detectable levels of EBV in PB. In the allo-HSCT group, 13 of 15 (87%) cases were EBV-associated either by virtue of EBV-positivity in the lymphoma (70%, n=7/10 analyzable patients), or EBV-positivity in PB (86%, n=12/14 analyzable patients). Involvement of the CNS was diagnosed in three of 25 patients (allo-HSCT, n=2; 13%; SOT, n=1; 10%).

### Latency from transplant to PTLD onset

**Figure 1:**
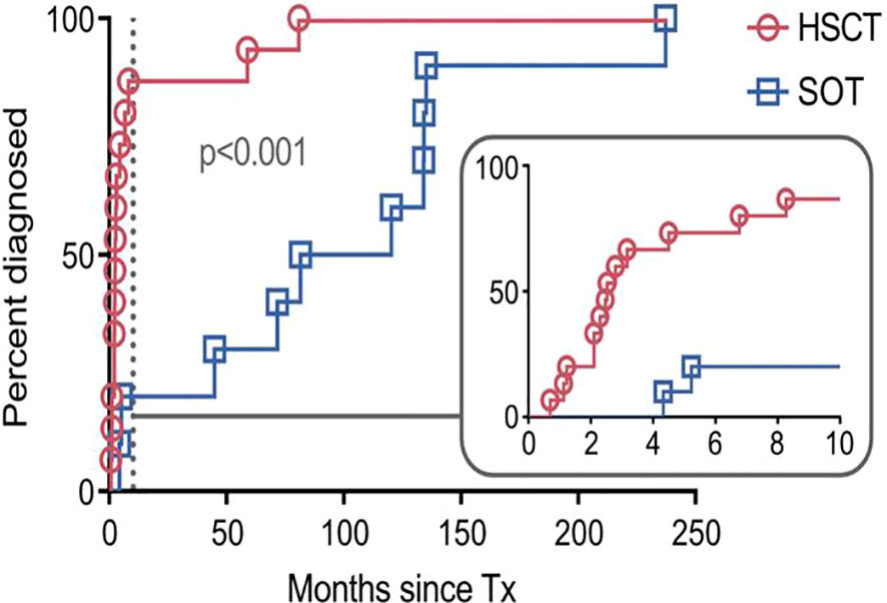
Time to diagnosis of post-transplant lymphoproliferative disorder (PTLD) after transplantation. Each symbol represents a PTLD diagnosis on a given day. The inset shows the same data up until month 10 (marked by dashed line) on an enlarged x-axis. SOT, recipients of solid organ transplantation; HSCT, recipients of hematopoietic stem cell transplantation; Tx, transplantation.

There was a strong difference in the latency period from transplantation to the occurrence of PTLD between the allo-HSCT group (median, 2 months; range, 0-81 months) as compared to the SOT group (median, 99 months; range, 4-233 months; P<0.001; **Figure 1**). In the allo-HSCT group, PTLD onset was strongly associated with ongoing immunosuppression: only one patient (HSCT14; **Table 1**) did not receive immunosuppressive therapy at the time of PTLD diagnosis. The second patient in the allo-HSCT group diagnosed >10 months after transplantation (HSCT9; **Table 1**) received long-term immunosuppressive therapy due to chronic graft-versus-host disease (GvHD).

### Patient characteristics at PTLD onset

In our cohort, ECOG-PS did not differ between the allo-HSCT and the SOT group (P=0.58; **Table 1**). In addition, Ann Arbor stages as well as the international prognostic index (IPI) of PTLD at diagnosis were similar between the two groups (**Table 1**).

### PTLD treatment and response

The most common first-line therapy was a switch in immunosuppression from cyclosporine A or tacrolimus to sirolimus or a rapid reduction of immunosuppression (both abbreviated ‘RIS’) in combination with rituximab +/- additional agents (RIS+R+X; **Table 2**). In the allo-HSCT group, 10 patients (66% of total; 77% of patients with CD20^+^ PTLD) received this form of first-line therapy. Of those, six (60%) responded with a CR and one (10%) patient responded with a partial response (PR). In the SOT group, eight patients (80%) received first-line RIS+R+X and 6 of them (75%) responded with a CR, while two patients (25%) responded with a PR.

**Table 2:** Treatment and response of patients with post-transplant lymphoproliferative disorders (PTLD) at onset of PTLD.

Patient ID	Treatment	Response	Follow- up (mo)^a^	Outcome	Cause of death
PTLD after HSCT:					
HSCT1	5xR, 3xEtoposide, IS-switch, 1xCHOP	refractory	0.5	fatal	PTLD
HSCT2	R->PD -> IS-switch, Ibrutinib -> CR	PD->CR	10.1	fatal	VZV-encephalitis
HSCT3	IS-switch, Panobinostat, Ibrutinib -> CR	CR	27.4	alive	
HSCT4	RIS, i.th. R/Cytarabine/MTX -> PR -> 2xMTX ->	PR -> CR	96.1	alive	
HSCT5	RIS, 5xR	refractory	2.2	fatal	MAS/PTLD
HSCT6	RIS, 6xR-MTX -> CR	CR	76.8	alive	
HSCT7	1xR	refractory	0.5	fatal	EBV-viremia/PTLD
HSCT8	RIS, 2xR & 1xEBV-T cells -> CR	CR	54.0	alive	
HSCT9	RIS, 6xGMALL-B-NHL 2002 (w/o anthracyclines) -> CR	CR	65.9	alive	
HSCT10	RIS, 1xR-CHOP 2xR	refractory	1.2	fatal	EBV-viremia/PTLD
HSCT11	RIS, 4xR -> CR	CR	7.4	alive	
HSCT12	RIS, 4xR -> CR -> 2xEBV-T cells -> CR	CR	0.9	alive	
HSCT13	RIS, 6xR-CHOP & 5xEBV-T cells -> CR	CR	9.2	alive	
HSCT14	(nolS), 2xAVD, RTx -> CR -> relapse -> 4xR, 9xBV, 1xDHAP, Nivolumab	CR-> relapse	67.2	alive	
HSCT15	RIS, 7xBV, DLI, RTx, 1xCHOP	refractory	12.0	fatal	PTLD
**Median**	**93% IS-switch/RIS^b^, 92%, R^b^, 23%VST^b^ 27% CHOP**	**67%CR**	**54.7 months**		
PTLD after SOT:					
SOT1	RIS, 4xR -> CR -> relapse	CR-> relapse	6.4	alive	
SOT2	RIS, 2xR-CHOP -> PR -> 4xR-CHOP ->PD -> 1xR-DHAP, 1xR-Pola-Benda	PR -> PD.	6.6	fatal	PTLD
SOT3	1xR-CHOP, 6xGMALL-B-NHL 2002 -> CR	CR	16.6	alive	
SOT4	RIS, RTx -> CR -> relapse -> 13xR-MTX	CR-> relapse	24.7	fatal	PTLD
SOT5	RIS, 1xR-CHOP -> CR	CR	33.2	alive	
SOT6	RIS, 12xR -> CR	CR	84.2	fatal	Colorectal cancer
SOT7	RIS, 4xR -> CR	CR	91.7	alive	
SOT8	RIS, 7xR-CHOP -> CR	CR	23.3	alive	
SOT9	RIS, 1xR, 6xR-Benda -> PR -> 1xR-CHOP	PR -> PD	11.2	fatal	Sepsis
SOT10	RIS, 8xR -> CR	CR	80.0	alive	
**Median**	**90% RIS, 100% R, 0%VST, 50% CHOP**	**80%CR,20%PR**	**33.7 months**		

^a^Since/at diagnosis of PTLD, respectively. ^b^Where applicable (*i.e.*, CD20^+^, EBV-associated, IS still ongoing).

AVD, adriamycin, vinblastine, dacarbazine; Benda, Bendamustine; BV, brentuximab-vedotin; CHOP, cyclophosphamide, doxorubicin, vincristine, prednisolone; DLI, Donor lymphocyte infusion; GMALL-B-NHL 2002, protocol containing R, Dexa, vincristine, MTX, ifosfamide, cytarabine, etoposide, cyclophosphamide, doxorubicin; IS, immunosuppression; Mito-FLAG, mitoxantrone, fludarabine, cytarabine, G-CSF; monom, monomorphic; MTX, Methotrexate; PD, Progressive disease; Pola, Polatuzumab-Vedotin; R, Rituximab; RIS, Reduction of immunosuppression; RTx, Radiotherapy; VST, Virus-specific T cells.

One patient of the allo-HSCT group (HSCT14) did not receive immunosuppressive therapy at the diagnosis of PTLD. Another patient (HSCT7) had severe chronic GvHD at PTLD onset and did, therefore, not undergo RIS. Two patients of the allo-HSCT group had CD20-negative PTLD subtypes (HSCT14, classic Hodgkin lymphoma [cHL] and HSCT15, T-lymphoblastic lymphoma [T-LBL]) and, therefore, did not receive rituximab. Treatment in the latter two cases consisted of doxorubicin, vinblastine, dacarbazine (AVD) + radiotherapy (RTx) or brentuximab-vedotin (BV) + donor lymphocyte infusions (DLI) + RTx + cyclophosphamide, doxorubicin, vincristine, prednisolone (CHOP), respectively.

Furthermore, many patients required chemotherapy. Five of 15 (33%) patients in the allo-HSCT group received anthracycline-containing regimens, of whom two (40%) achieved a CR. In the SOT group, 5 of 10 (50%) patients received anthracyclines of whom three (60%) achieved a CR and two (40%) achieved a PR.

Finally, three of 13 (23%) patients in the allo-HSCT group with EBV detection in their lymphoma and/or PB received virus-specific T cells (VST). All of them are in ongoing CR. None of the patients in the SOT group received VST.

**Figure 2:**
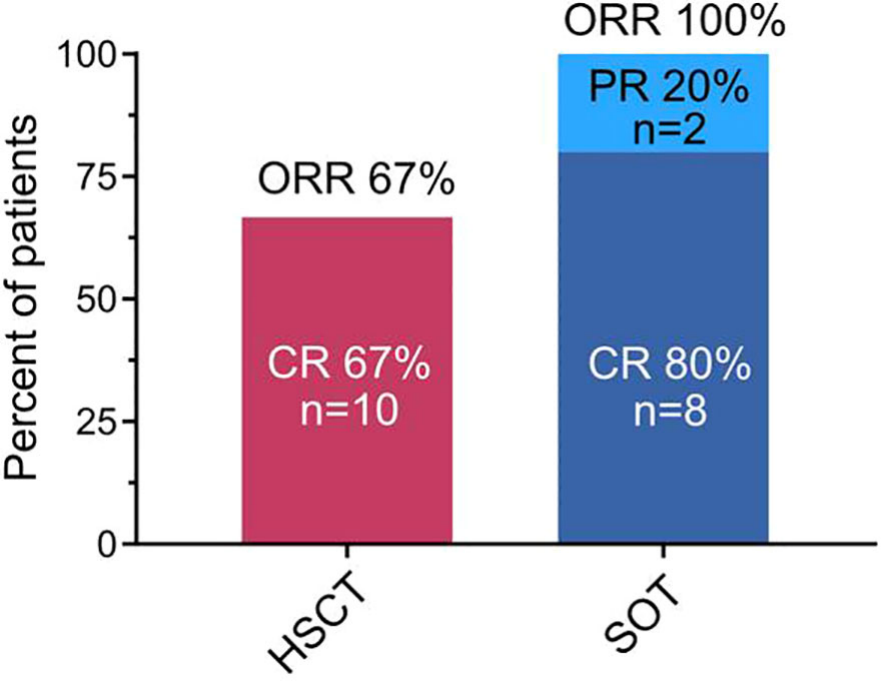
Overall response rate (ORR) as characterized by best response to treatment. Overall response rate included patients with achievement of complete response or partial response to any treatment after the diagnosis of post-transplant lymphoproliferative disorder. Percentages and numbers of patients achieving complete response (CR), partial response (PR), and that of the overall response rate are indicated group-wise. SOT, recipients of solid organ transplantation; HSCT, recipients of hematopoietic stem cell transplantation.

The best response to treatment at any time was aggregated into an overall response rate (ORR; including CRs and PRs). This ORR was 67% (n=10/15) in the allo-HSCT group and 100% (n=10/10) in the SOT group (**Figure 2**). Of note, all responders in the allo-HSCT group achieved a CR. Eighty percent (n=8/10) of SOT patients achieved a CR and 20% (n=2/10) achieved a PR.

**Figure 3:**
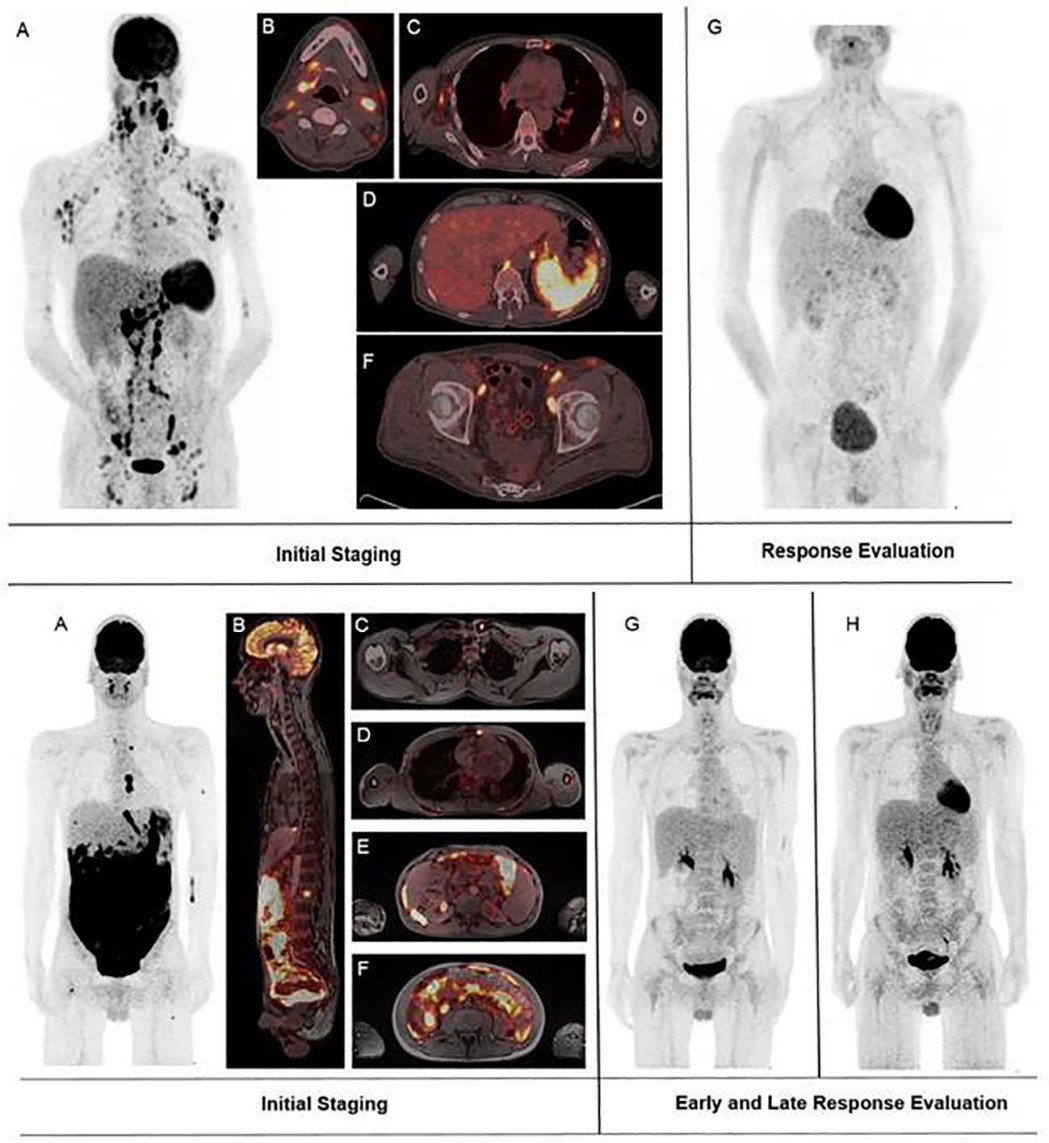
Representative fluorodeoxyglucose positron emission tomography–computed tomography and magnetic resonance tomography (^18^F-FDG PET/CT and MRT) images of two patients who achieved a complete response. Upper panel: **(A–F)** ^18^F-FDG PET/CT of patient HSCT12 on the day of diagnosis of post-transplant lymphoproliferative disorder (PTLD). **(G)** ^18^F-FDG PET/CT of the same patient 49 days after PTLD diagnosis. The patient had received a 100% reduction of their immunosuppression and 4 cycles of rituximab in the meantime. Lower panel: **(A–H)** ^18^F-FDG PET/MRT of patient SOT3 on the day of start of PTLD treatment. **(G)** ^18^F-FDG PET/MRT of the same patient 44 days after start of PTLD treatment. The patient had received one cycle of R-CHOP and one cycle of intensive anthracycline-based chemotherapy in the meantime. **(H)** ^8^F-FDG PET/MRT of the same patient 151 days after start of PTLD treatment.

^18^F-FDG-PET for metabolic response assessment was available in 6 (HSCT9, HSCT12, HSCT14, HSCT15, SOT1, and SOT3) of the 25 patients. Of these, 5 had a complete metabolic response (CMR; Deauville 1-3) and one (HSCT15) had a morphologically stable disease with a partial metabolic response (PMR; Deauville 4-5). Representative ^18^F-FDG-PET/CT and -MR images of two patients who achieved CMR (Deauville 2) are shown in **Figure 3**.

### Relapse-free and overall survival

**Figure 4:**
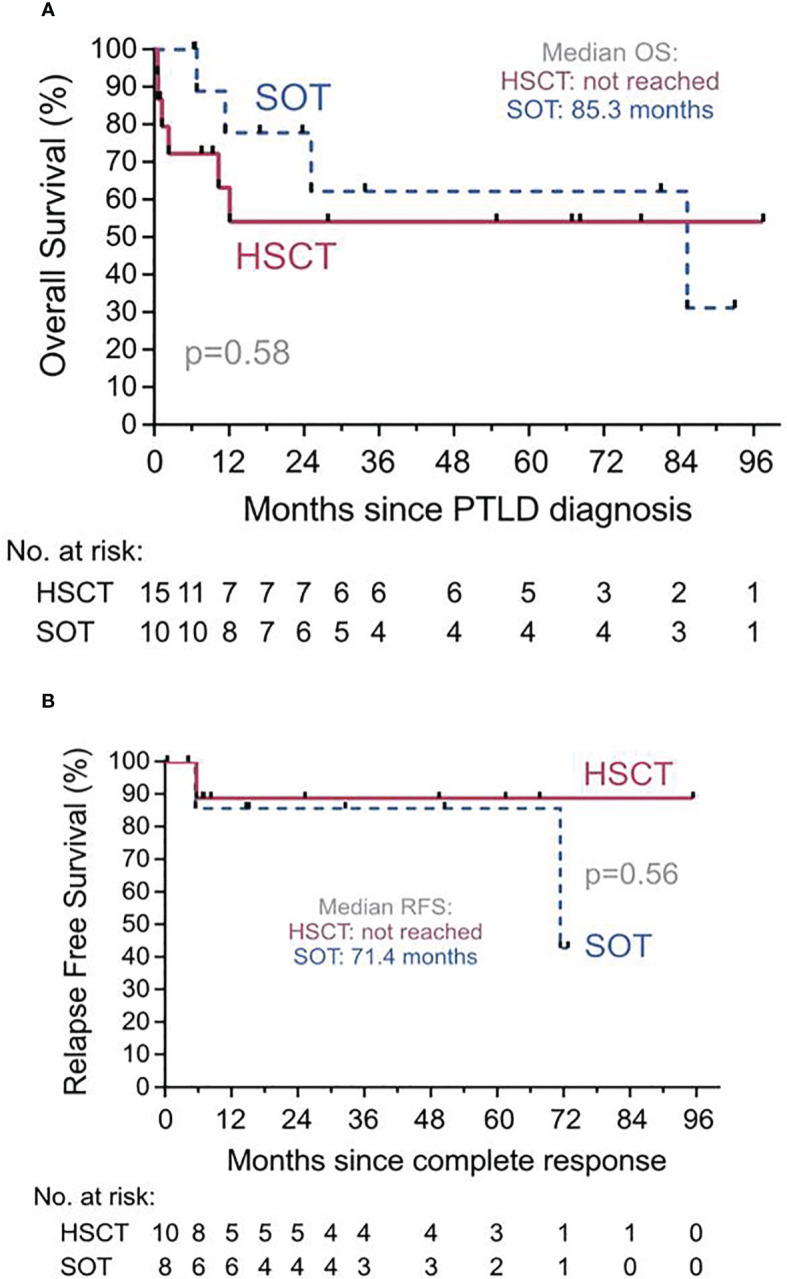
Overall Survival (OS) and Relapse Free Survival (RFS). **(A)** OS from diagnosis of PTLD for recipients of solid organ transplantation (SOT, dashed line) or of hematopoietic stem cell transplantation (HSCT, solid line). Median OS for allo-HSCT was not reached. PTLD, Post transplant lymphoproliferative disorder. **(B)** RFS since complete response for recipients of SOT (dashed line) or of allo-HSCT (solid line) who achieved a complete response of their PTLD at any point. Median OS for allo-HSCT was not reached.

Data on response and outcome were available in all patients. Median follow-up after diagnosis of PTLD was 54.7 months (range, 0.5-97.5 months) after allo-HSCT and 33.7 months (range, 6.5-93.0 months) after SOT. Median OS was not reached for allo-HSCT patients and was 85.3 months for SOT patients. The estimated 1-year RFS and OS were 89% (95%-CI, 43-98%) and 54% (95%-CI, 24-77%) in the allo-HSCT and 86% (95%-CI, 33-98%) and 78% (95%-CI, 36-94%) in the SOT cohort, respectively (**Figure 4**).

One patient in the allo-HSCT and two patients in the SOT group relapsed after achievement of CR of the PTLD, respectively. Six (40%) patients died in the allo-HSCT group, (relapse of PTLD, n=5; VZV-encephalitis n=1) and 4 (40%) patients in the SOT group (relapse of PTLD, n=2; colorectal cancer n=1; sepsis, n=1).

### Prognostic factors for overall survival

In univariate analysis, time from transplantation to PTLD onset ≤150 days was significantly associated with a lower OS in the allo-HSCT group only (P=0.046). In the SOT group, ECOG-PS >2 was identified as significant prognostic factor of a lower OS (P=0.03). Age and IPI were not significantly associated with OS in either of the groups (**Table 3**).

**Table 3:** Univariate analysis of prognostic factors for overall survival in patients with post-transplant lymphoproliferative disorders after hematopoietic stem cell or solid organ transplantation.

Variables	allo-HSCT		SOT	
	Patients, n (%)	P-value	Patients, n (%)	P-value
Age (years)				
>60	6 (40)	0.17	3 (30)	0.9
≤60	9 (60)		7 (70)	
ECOG-PS				
>2	7 (47)	0.2	2 (20)	0.03
≤2	8 (53)		8 (80)	
IPI				
>2	10 (77)	0.24	8 (80)	0.94
≤2	3 (23)		2 (20)	
PTLD onset (post-Tx)				
>150 days	4 (27)	0.046	9 (90)	0.94
≤150 days	11 (73)		1 (10)	

ECOG-PS, Eastern Cooperative Oncology Group performance status; IPI, international prognostic index; PTLD, post-transplant lymphoproliferative disorder.

## Discussion

The focus of our study was to characterize adult patients with PTLD after allo-HSCT as well as SOT in a cohort study from three University hospitals and to compare differential outcomes.

Latency from transplantation to the onset of PTLD was shorter in the allo-HSCT group, similar to what is reported in the literature (11, 18). This is likely due to the strong immunosuppressive therapy after allo-HSCT. Both, the extent and duration of the immunosuppressive therapy are main risk factors for development of PTLD (2). This is corroborated by the fact that almost all patients of the allo-HSCT group were still on immunosuppressive therapy at the time of PTLD diagnosis. Tailored maintenance immunosuppressive regimens, however, may help to reduce the risk of PTLD onset (19).

In contrast to other reports (3), we provide comprehensive clinical characteristics including ECOG-PS, stage, and IPI at the time of PTLD diagnosis for allo-HSCT patients. Patient characteristics at time of PTLD diagnosis were comparable between both groups. Main differences were the frequency of malignancies as the indication for the transplantation and, in conjunction with that, the fraction of patients that previously received anthracycline-containing treatments. Surprisingly, this higher burden of pretreatment with chemotherapy and lower latency in the allo-HSCT group did not translate into a lower median ECOG-PS. There was, however, a larger fraction of patients with ECOG-PS >2. An ECOG-PS ≤2 was a prerequisite for inclusion in the phase II PTLD-1 trial (NCT01458548) and the phase II PTLD-2 trial (NCT02042391). These trials exclusively recruited patients with PTLD after SOT and demonstrated that a risk-stratified sequential treatment with rituximab +/- CHOP is safe and effective (10, 11). However, the higher fraction of patients with ECOG-PS >2, the high susceptibility to cytopenias after chemotherapy, and the common previous exposure to anthracyclines warrants caution when transferring the results of the safety of the CHOP regimen from these trials to patients with PTLD after allo-HSCT.

The reported treatment strategies of PTLD in our series involved RIS or IS-switch and rituximab in almost all cases in both groups. RIS is an established approach in PTLD after SOT and has been shown to be an independent prognostic factor in PTLD after allo-HSCT (4). However, RIS after allo-HSCT bears the risk of promoting GvHD and can be prohibitive in cases of coexisting extensive active GvHD, such as in one of our cases (HSCT7). How to balance the need for both decreased IS for the treatment of PTLD and increased IS for treatment of GvHD in such cases remains to be elucidated. The combined immunosuppressive and potential anti-lymphoma effects by mTOR inhibitors (i.e. Sirolimus, Everolimus) are hypothesized, but not systematically validated, rationales.

Fewer patients in the allo-HSCT group received CHOP regimens as compared to the SOT group. The relatively low percentage reflects the high prevalence of prior anthracycline-containing therapies for malignancies in the allo-HSCT group (60%). For example, concerns about the level of cumulative anthracycline doses are documented as reasons to omit them in the treatment of PTLD in HSCT9. Therefore, prior treatment with chemotherapy and cumulative toxicities are a major consideration when choosing PTLD treatments in this group.

EBV-specific T-cell therapy (VST) was used exclusively in allo-HSCT patients. However, adoptive transfer of EBV-specific T cells has shown great promise for the treatment of EBV-viremia and EBV^+^ PTLD in SOT as well (20, 21). We suspect that, because VST are an established treatment for viral infections after allo-HSCT, physicians were more experienced and inclined to provide VST to allo-HSCT PTLD patients. Moreover, the greater use of VST in the allo-HSCT group was probably due to the markedly higher EBV-association. Other works that defined EBV-positivity as the histological finding of EBV-encoded small RNAs (EBERs) have also found the frequency of EBV-positivity to be ~83% after allo-HSCT (3) and to be ~60% in a SOT dominated cohort (22). In our SOT cohort, only two of four patients with EBER-positive PTLD also had detectable EBV levels in the peripheral blood (PB). Data on the comparative detection of EBV genetic material in PTLD lesions and PB are lacking. Thus, EBER tests should always be performed so as not to overlook EBV-association and miss out on VST treatment options. However, EBV-positive PTLD can also develop in the absence of high viral loads. In addition, high viral loads are not predictive in all settings, and a decline in viral loads may not always predict treatment response. Nevertheless, a rapid decline in EBV load occurs almost invariably after anti-B cell monoclonal antibody treatment, irrespective of long term PTLD response (23). The lack of standardization of techniques and results for assessment of circulating EBV DNA has been an additional complicating factor. Therefore, an international WHO standard for determining EBV load was published in 2013 (23).

With the approval of tabelecleucel for relapsed or refractory EBV-associated PTLD after SOT or allo-HSCT (20), we expect this form of therapy to become more widespread.

Beyond these options, patients in both groups received a wide variety of treatments. These were usually chosen to address the specific subtype of PTLD: two patients (HSCT6 and SOT4) received local therapy and methotrexate for PTLD, while three patients received regimens that were more akin to those developed for the non-PTLD entity counterparts (HSCT9 with ‘GMALL-B-NHL 2002’ for Burkitt lymphoma, HSCT14 with AVD + RTx for cHL, HSCT15 with BV for T-cell PTLD). This aspect is often underreported in PTLD studies. PTLD subtypes themselves appear to be prognostic factors for OS (24–26). However, the heterogeneity in treatments even within PTLD subtypes hampers the identification of prognostic factors and optimal treatment algorithms from retrospective analyses. In addition, there is a concern that chemotherapy might result in relapses of original hematological malignancies or graft rejection. Therefore, more prospective studies are warranted towards a PTLD-specific prognostic model.

In our cohort, the ORR was lower in the allo-HSCT group. Consequently, the OS trended towards an inferior outcome for this group. However, a substantial fraction of patients in the SOT group eventually experienced progressive PTLD. Nevertheless, our data compare favorably with other published analyses (11, 27, 28). In the allo-HSCT group, time from transplantation ≤150 days was identified as a prognostic factor for lower OS in a univariate analysis. PTLD onset as a prognostic factor was observed to varying degrees in previous works (4, 10). One study by the Infectious Diseases Working Party of the European Group for Blood and Marrow Transplantation found a trend for lower OS for PTLD onset <100 days after allo-HSCT (4) and another found that later onset of PTLD after SOT had a longer time to progression (10). In our analysis on the total PTLD cohort, neither age, ECOG-PS or IPI were associated with lower OS. In the SOT group, however, ECOG-PS > 2 was significantly associated with lower OS. The low patient numbers of our cohort were prohibitive of a multivariable analysis and warrant caution when interpreting the significance of the identified prognosticators.

Only a very small number of studies directly compared cases of PTLD after allo-HSCT with that after SOT (29–32). These found a later onset, a higher ORR to treatment as well as a trend for a longer OS in the SOT group as compared to the allo-HSCT group. In contrast, the relapse rate was higher in the SOT as compared to the allo-HSCT group (24, 25, 27, 28). However, in most of these studies treatment is not comprehensively reported or patients from the pre-rituximab era are included. Particularly, in these series rituximab was given to only 36% (n=10) of PTLD patients after SOT and 38% (n=5) of PTLD patients after allo-HSCT (29, 31). This presumably influenced the rather poor median OS in the allo-HSCT groups of <12 months in the majority of studies. Therefore, our study provides important data on more recently treated PTLD patients.

Limitations of this study included its retrospective nature, the small size of the cohorts, and the low number of patients who received a PET-based response assessment. Recently, novel insights into pathogenesis (33) and treatment strategies for PTLD have emerged, including adoptive immunotherapy and targeted therapeutics, such as tabelecleucel, CAR-T cells, and CD30-based therapy (34), that could substantially improve outcomes.

In the near future, data from the phase-3 ALLELE study (NCT03394365) testing tabelecleucel vs. placebo in patients with PTLD after allo-HSCT or SOT might yield more insight into differential response patterns in a relapsed or refractory setting.

In conclusion, we show that PTLD after allo-HSCT has an earlier onset, a lower ORR, and tends to have a worse outcome when compared to PTLD after SOT in a multicenter cohort. We highlight that GvHD and pre-exposure to anthracyclines are important considerations in the treatment of PTLD after allo-HSCT and describe existing treatment heterogeneities. Refined prognostic models are needed. Finally, novel therapies for PTLD are being approved and new studies are warranted to analyze their safety and efficacy in a real-world setting.

## Data availability statement

The datasets presented in this article are not readily available because The raw data supporting the conclusions of this article are restricted in accordance with the local legislation. Requests to access the datasets should be directed t\o s.kayser@dkfz-heidelberg.de.

## Ethics statement

Ethical review and approval was not required for the study on human participants in accordance with the local legislation and institutional requirements. The patients/participants provided their written informed consent to participate in this study.

## Author contributions

PL and SK were responsible for the concept of this paper, contributed to the literature search and data collection, analysed and interpreted data, and wrote the manuscript. PL, AR, UH, MH, UP and SK contributed patients and critically revised the manuscript. AM and LK performed research and critically revised the manuscript. All authors contributed to the article and approved the submitted version.
