# Erratum: Characterization and outcome of post-transplant lymphoproliferative disorders within a collaborative study

**DOI:** 10.3389/fonc.2023.1290218

**Published:** 2023-09-20

**Authors:** Frontiers Production Office

**Affiliations:** Frontiers Media SA, Lausanne, Switzerland

**Keywords:** PTLD, Epstein - Barr virus, allogeneic hematopoietic stem cell transplantation, solid organ transplantation, outcome

Due to a production error, there was a mistake in the footnote for **Table 1** as published. The abbreviations were written as ‘^a^Since/at diagnosis of PTLD, respectively. ^b^Where applicable (i.e., CD20+, EBV-associated, IS still ongoing).

7 + 3, cytarabine and daunorubicin; (s)AML, (secondary) acute myeloid leukemia; Assoc, associated; sAA, severe aplastic anemia; AVD, adriamycin, vinblastine, dacarbazine; Benda, Bendamustine; BV, brentuximab-vedotin; CAD, cyclophosphamide, doxorubicin, and dexamethasone; cALL, common acute lymphoblastic leukemia; CHOP, cyclophosphamide, doxorubicin, vincristine, prednisolone; cHL, classic Hodgkin lymphoma; CKD, chronic kidney disease; CNL, Chronic neutrophilic leukemia; DLBCL, diffuse large B-cell lymphoma; DLI, Donor lymphocyte infusion; GMALL07/2003, study protocol containing daunorubicin and doxorubicin; GMALL-B-NHL 2002, protocol containing R, Dexa, vincristine, MTX, ifosfamide, cytarabine, etoposide, cyclophosphamide, doxorubicin; HCC, Hepatocellular carcinoma; IDA-FLAG, idarubicin, fludarabine, cytarabine, G-CSF; IS, immunosuppression; Mito-FLAG, mitoxantrone, fludarabine, cytarabine, G-CSF; Monom, monomorphic; MTX, Methotrexate; PBL, lasmablastic lymphoma; PCL, plasma cell leukemia; PD, Progressive disease; Pola, Polatuzumab-Vedotin; PSC, Primary sclerosing cholangitis; R, Rituximab; RIS, Reduction of immunosuppression; RTx, Radiotherapy; T-LBL, Tlymphoblastic lymphoma; T1D, Type 1 diabetes; TAD, thalidomide, doxorubicin, Dexa; T-ALL, T-acute lymphoblastic leukemia; VST, Virus-specific T cells. Values in bold means the summarized values.’

The correct footnote appears below. The publisher apologizes for this mistake.

‘ ^a^Since/at diagnosis of PTLD, respectively. ^b^Where applicable (*i.e.*, CD20^+^, EBV-associated, IS still ongoing).

Abbreviations: 7 + 3=cytarabine and daunorubicin. (s)AML=(secondary) acute myeloid leukemia. assoc.=associated. sAA=severe aplastic anemia. CAD=cyclophosphamide, doxorubicin, and dexamethasone. cALL=common acute lymphoblastic leukemia. cHL=classic Hodgkin lymphoma. CKD=chronic kidney disease. CNL=Chronic neutrophilic leukemia. DLBCL=diffuse large B-cell lymphoma. GMALL07/2003=study protocol containing daunorubicin and doxorubicin. HCC=Hepatocellular carcinoma. IDA-FLAG=idarubicin, fludarabine, cytarabine, G-CSF. Mito-FLAG=mitoxantrone, fludarabine, cytarabine, G-CSF. monom.=monomorphic. PBL=Plasmablastic lymphoma. PCL=plasma cell leukemia. PD=Progressive disease. Pola=Polatuzumab-Vedotin. PSC=Primary sclerosing cholangitis. T-LBL=T-lymphoblastic lymphoma. T1D=Type 1 diabetes. TAD=thalidomide, doxorubicin, Dexa. T-ALL=T-acute lymphoblastic leukemia.’

The original version of this article has been updated.

Additionally, due to a production error, there was a mistake in the footnote for Table 2 as published. The abbreviations were written as ‘ ^a^Since/at diagnosis of PTLD, respectively. ^b^Where applicable (i.e., CD20+, EBV-associated, IS still ongoing).

AVD, adriamycin, vinblastine, dacarbazine; Benda, Bendamustine; BV, brentuximab-vedotin; CAD, cyclophosphamide, doxorubicin, and dexamethasone; CHOP, cyclophosphamide, doxorubicin, vincristine, prednisolone; DLBCL, diffuse large B-cell lymphoma; DLI, Donor lymphocyte infusion; GMALL07/2003, study protocol containing daunorubicin and doxorubicin; GMALL-B-NHL 2002, protocol containing R, Dexa, vincristine, MTX, ifosfamide, cytarabine, etoposide, cyclophosphamide, doxorubicin; HCC, Hepatocellular carcinoma; IDA-FLAG, idarubicin, fludarabine, cytarabine, G-CSF; IS, immunosuppression; Mito-FLAG, mitoxantrone, fludarabine, cytarabine, G-CSF; monom;, monomorphic; MTX, Methotrexate; PD, Progressive disease; Pola, Polatuzumab-Vedotin; PSC, Primary sclerosing cholangitis; R, Rituximab; RIS, Reduction of immunosuppression; RTx, Radiotherapy; thalidomide, doxorubicin, Dexa; VST, Virusspecific T cells. Values in bold means the summarized values.’

The correct footnote appears below. The publisher apologizes for this mistake.

^‘a^Since/at diagnosis of PTLD, respectively. ^b^Where applicable (*i.e.*, CD20^+^, EBV-associated, IS still ongoing).

AVD, adriamycin, vinblastine, dacarbazine; Benda, Bendamustine; BV, brentuximab-vedotin; CHOP, cyclophosphamide, doxorubicin, vincristine, prednisolone; DLI, Donor lymphocyte infusion; GMALL-B-NHL 2002, protocol containing R, Dexa, vincristine, MTX, ifosfamide, cytarabine, etoposide, cyclophosphamide, doxorubicin; IS, immunosuppression; Mito-FLAG, mitoxantrone, fludarabine, cytarabine, G-CSF; monom, monomorphic; MTX, Methotrexate; PD, Progressive disease; Pola, Polatuzumab-Vedotin; R, Rituximab; RIS, Reduction of immunosuppression; RTx, Radiotherapy; VST, Virus-specific T cells.’

Due to a production error, there was a mistake in **Table 1** as published. ‘IDA-FLAG’ was incorrectly written as ‘DA-FLAG’, ‘Alcoholic cirrhosis’ was incorrectly written as ‘alcoholic cinhosis’, and ‘‘Multiple Myeloma’ was incorrectly written as ‘Myelom’. The corrected **Table 1** appears below. The publisher apologizes for this mistake.

**Table 1:** 

Patient ID	Age^a^	Sex	Tx type	Indication for Tx	Previous anthracycline- containing treatments	Conditioning	Time from Tx (months/yrs)^a^	Histology^a^	EBV+ (Lymphoma/Blood)	Ann Arbor stage^a^	ECOG^a^	IPI^a^
PTLD after HSCT:												
HSCT1	36	m	2nMMUD	sAML	1x 7 + 3, 1x IDA-FLAG	MA	2(0.2)	monom, DLBCL	pos/pos	n/a	3	n/a
HSCT2	65	m	MUD	AML	1x 7 + 3, 1x HAM	NMA	2(0.2)	n/a	na/pos	.	4	4
HSCT3	46	m	MUD	sAA	none	NMA	0(0)	n/a	na/pos	.	3	3
HSCT4	59	m	MUD	PCL	1x CAD	NMA	6(0.5)	n/a	na/pos	IV	3	3
HSCT5	61	m	MMUD	AML	2x 7 + 3, 1x HAM	MA	2(0.2)	n/a	na/pos	n/a	3	n/a
HSCT6	32	m	MUD	sAA	none	NMA	8(0.7)	monom, DLBCL	pos/pos	I	1	0
HSCT7	64	W	2^nd^ MUD	CNL	1x Mito-FLAG, 1x IDA- FLAG	NMA	4(0.3)	monom, DLBCL	pos/pos	IV	3	4
HSCT8	48	W	MUD	cALL	treatment within the GMALL 07/2003 trial	MA	2(0.2)	monom, MM	neg/pos	IV	3	3
HSCT9	64	m	AMMUD	sAML	2x 7 + 3	NMA	58(4.8)	monom Burkitt	neg/neg	IV	1	3
HSCT10	60	m	MUD	sAA	none	MA	1(0.1)	polymorphic	pos/pos	IV	2	3
HSCT11	61	m	MUD	sAA	none	MA	1(0.1)	n/a	na/pos	I	1	2
HSCT12	57	m	2nd MUD	T-ALL&AML	1x 7 + 3	MA	2(0.2)	monom, DLBCL	pos/pos	IV	2	3
HSCT13	47	m	MUD	Metachromatic leukodystrophy	none	NMA	2(0.2)	monom, DLBCL	pos/pos	.	2	3
HSCT14	50	w	MUD	Multiple Myeloma	3x TAD, 1x CAD	NMA	81 (6.7)	cHL	pos/na	I	0	0
HSCT15	67	w	MRD	MDS EB2	none	NMA	3(0.3)	monom., T- LBL	neg/neg	IV	2	5
Median/%	59	73%	93% U	67% malignancies	60% anthracyclines		2(0.17)	80% monom.	87% assoc.	IV	2	3
PTLD after SOT:												
SOT1	53	W	Kidney	CKD, Fabry disease			133(11.1)	monom, DLBCL	neg/na	I	1	0
SOT2	31	m	Kidney	Monolateral renal agenesis			118(9.8)	monom, DLBCL	neg/neg	IV	3	3
SOT3	29	m	Liver	PSC, autoimmune hepatitis			70 (5.8)	monom, DLBCL	pos/neg	IV	1	3
SOT4	74	W	Liver	Autoimmune hepatitis			80 (6.7)	polymorphic	pos/pos	IV	3	4
SOT5	67	W	Kidney	CKD, chr. glomerulonephritis			233 (19.4)	monom, DLBCL	neg/na	IV	2	4
SOT6	57	m	Liver	Alcoholic cirrhosis			4 (0.3)	monom, PBL	pos/pos	I	2	1
SOT7	54	m	Liver	HCC, alcoholic cirrhosis			44 (3.7)	monom, DLBCL	neg/neg	IV	2	3
SOT8	67	m	Liver	Alcoholic cirrhosis			132 (11)	monom, DLBCL	neg/na	I	2	3
SOT9	45	m	Kidney & Pancreas	CKD, T1D			132 (11)	polymorphic	neg/neg	IV	2	4
SOT10	48	W	Liver	Alcoholic cirrhosis			5 (0.4)	polymorphic	pos/neg	IV	2	4
Median/%	54	60%/ m		10% malignancies			99 (8.3)	70% monom.	40% assoc.	IV	2	3

